# Complete sequence-based pathway analysis by differential on-chip DNA and RNA extraction from a single cell

**DOI:** 10.1038/s41598-017-10704-4

**Published:** 2017-09-08

**Authors:** D. van Strijp, R. C. M. Vulders, N. A. Larsen, J. Schira, L. Baerlocher, M. A. van Driel, M. Pødenphant, T. S. Hansen, A. Kristensen, K. U. Mir, T. Olesen, W. F. J. Verhaegh, R. Marie, P. J. van der Zaag

**Affiliations:** 1 Philips Research Laboratories, High Tech Campus 11, 5656 AE Eindhoven, The Netherlands; 2 Philips BioCell, Gydevang 42, 3450 Lillerød, Denmark; 3 Fasteris SA, Chemin du Pont-du-Centenaire 109, CH-1228 Plan-les-Ouates, Switzerland; 4 DTU Nanotech, Ørsteds Plads Building 345 east, 2800 Kgs, Lynby, Denmark; 5 XGenomes, Pagliuca Harvard Life Lab, 127 Western Avenue, Boston, MA 02134 USA

## Abstract

We demonstrate on-chip, differential DNA and RNA extraction from a single cell using a microfluidic chip and a two-stage lysis protocol. This method enables direct use of the whole extract, without additional washing steps, reducing sample loss. Using this method, the tumor driving pathway in individual cells from a colorectal cancer cell line was determined by applying a Bayesian computational pathway model to sequences obtained from the RNA fraction of a single cell and, the mutations driving the pathway were determined by analyzing sequences obtained from the DNA fraction of the same single cell. This combined functional and mutational pathway assessment of a single cell could be of significant value for dissecting cellular heterogeneity in tumors and analyzing single circulating tumor cells.

## Introduction

Accumulation of genetic changes leads to inter-cellular heterogeneity within tumours producing sub-populations of cells that have different potentials for metastasis and drug resistance^1^. Molecular differences between cells can be investigated by transcriptome^2, 3^ and genome^4, 5^ sequencing. We have developed a novel microfluidic device and a two-stage cell lysis method to extract and sequence separately both RNA and DNA from the same single cell. We applied this to cells from colorectal cancer cell lines to show, using a Bayesian computational pathway model^6^, that Wnt transcriptional pathway activity could be derived from the obtained single cell RNA profile. Whole-genome sequence data from the same cell revealed which genes in the Wnt pathway were mutated and hence candidate drivers of the aberrant signalling. Such single-cell resolved molecular phenotype to genotype correlations have implications in cancer management, including prognosis, prediction and personalized treatment.

Sequencing tumours in routine clinical practice reveals a multitude of variants, whose relevance for the individual patient is difficult to assess. This has led to discussions about driver and passenger mutations^1^ and with the premise that cancer is a clonal evolutionary process of somatic mutations^7^, the current focus is on known actionable mutations^8^. Unfortunately, a direct relationship does not always exist between DNA mutations and the activated molecular pathways driving tumor growth. In medulloblastoma, a direct relationship exists between mutations in *CTNNB1* (coding for ß-catenin) and Wnt activity. In liver carcinoma such a clear relationship between *CTNNB1* mutations and the Wnt pathway activation does not exist^6^, while in breast cancer, a mutation in *PIK3CA* is not always predictive of PI3K inhibitor response^9^. Epigenetic changes may also activate molecular pathways. We propose to identify driver mutations by determining both the genotype and the molecular phenotype^10^ of a tumor^6, 11, 12^: mRNA patterns will reveal *which* pathway is activated and DNA mutations will reveal *where* within the pathway the deregulation occurs. Thus, druggable mutations that might be the root cause of the activated pathway can be found. An ability to conduct such genotype and molecular phenotype analysis on single cells provides the resolution needed to address heterogeneity within a tumour and the sensitivity for a similar analysis on circulating tumour cells (CTCs).

Two recent approaches describe analysing the genome and transcriptome from the same single cell: DR-seq (gDNA-mRNA sequencing)^13^ and G&T-seq (genome and transcriptome sequencing)^14^. Both are bench protocols with multiple handling steps, not ideal in a diagnostic setting and risking sample loss. We report a two-step lysis procedure on cells captured in picoliter traps in a microfluidic chip, to extract consecutively the RNA and DNA from a single cell. This simplifies processing of the extracts and enables the use of conventional, commercial kits for single cell RNA and DNA amplification on the separated nucleic acid fractions. After sequencing both extracts, we assess complete pathway activity at both geno- and phenotypical level in single cells using a Bayesian network-based computational pathway model previously applied only to bulk samples^6, 12^.

## Results

**Figure 1:**
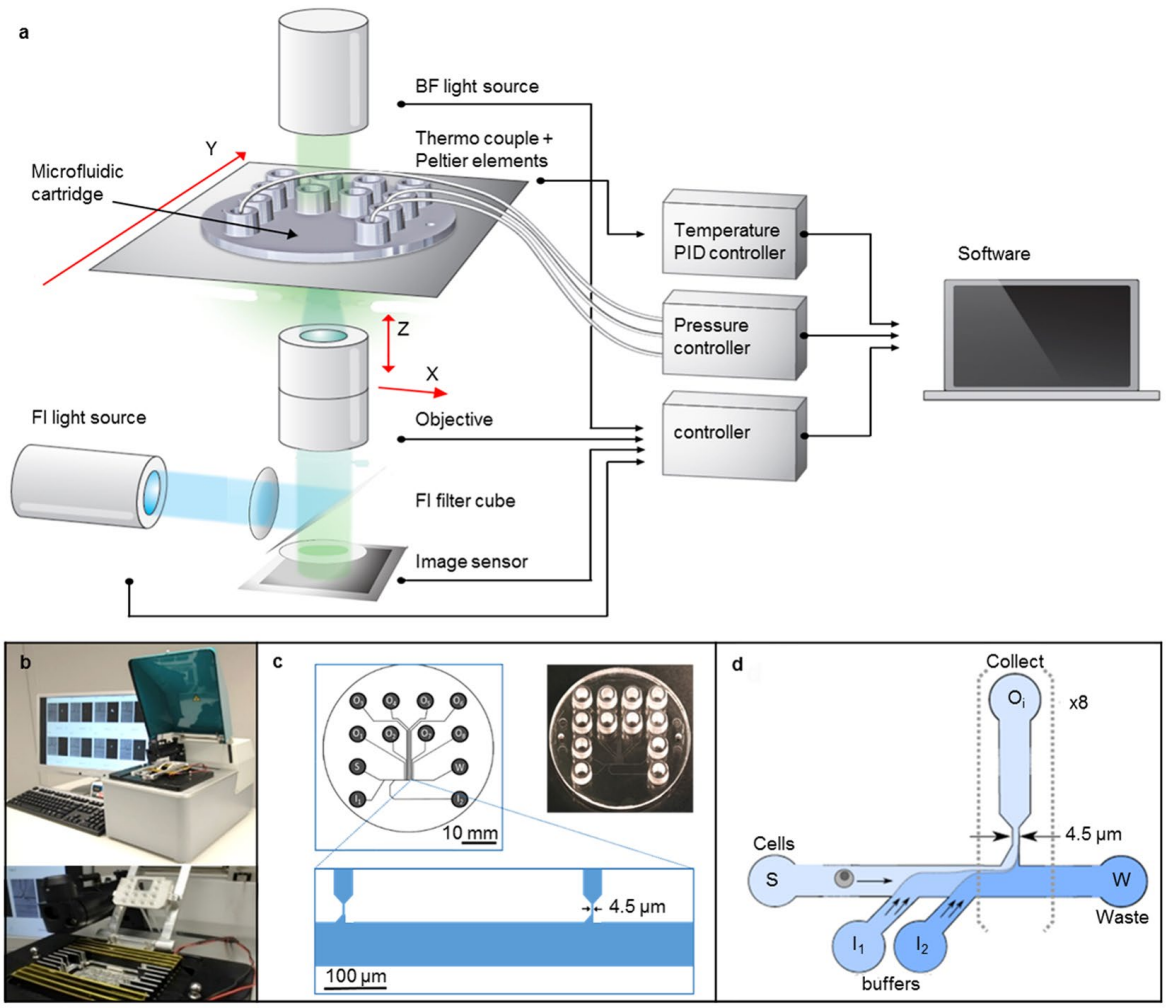
Single cell microfluidics instrument and chip. (**a**) Diagram and (**b**) picture of the set-up for operating the single cell platform. The system is a modified Philips BioCell FluidScope and provides bright field (BF) and epifluorescence (FL) *in-situ* monitoring of cells and their processing, a stage to mount microfluidic chips, focus automation and pressure-driven flow control (for details see Methods). A separate heating and cooling module in the set-up (see lower half Fig. 1b) enables nucleic acid amplification within the chip. The lower half of (**b**) shows also the position in which the microfluidic chip is mounted and the gasket through which pressure-driven flow is applied. (**c**) Diagram and picture of the single-use injection-molded microfluidic chip. In the diagram S labels the sample input, I_1_ and I_2_ the two buffer inputs, O_*i*_ the in total 8 outputs and W the waste chamber. The insert shows a blow-up of the channel and two of the 8 traps. (**d**) Schematic diagram of how cells are aligned in the main channel by flow-pinching and delivered to the traps downstream.

To sequence both RNA and DNA from the same single cell, we use a microfluidic chip to process cells and their content, plus a two-stage lysis procedure where the plasma membrane is lysed first to release the cytoplasmic RNA and then the nuclear membrane is lysed to release the DNA. A microfluidic chip is mounted in a set-up capable of affecting cell flow and monitoring individual cells and their processing (Fig. 1). Figure 1a gives a schematic diagram of the system, while Fig. 1b shows pictures. The system provides bright field and fluorescence *in-situ* monitoring of the cells and allows them to be processed in the chip by stepwise flow of reagents. The movement of the cells and reagent liquids in the chip is controlled by a pressure-driven system. A heating and cooling stage enables thermal cycling of the chip for nucleic acid amplification. Figure 1c shows the layout and a picture of the chips used. The pressure-driven microfluidics enables a valveless, passive, inexpensive, disposable chip to perform all necessary functions. Figure 1d shows how PBS buffer applied from the other inlet channels I_1_ and I_2_ pushes the cells, flowing from inlet S to the waste chamber, in the direction of the traps, each connected to individual outlets (O_*i*_ with *i* being 1 to 8).

**Figure 2:**
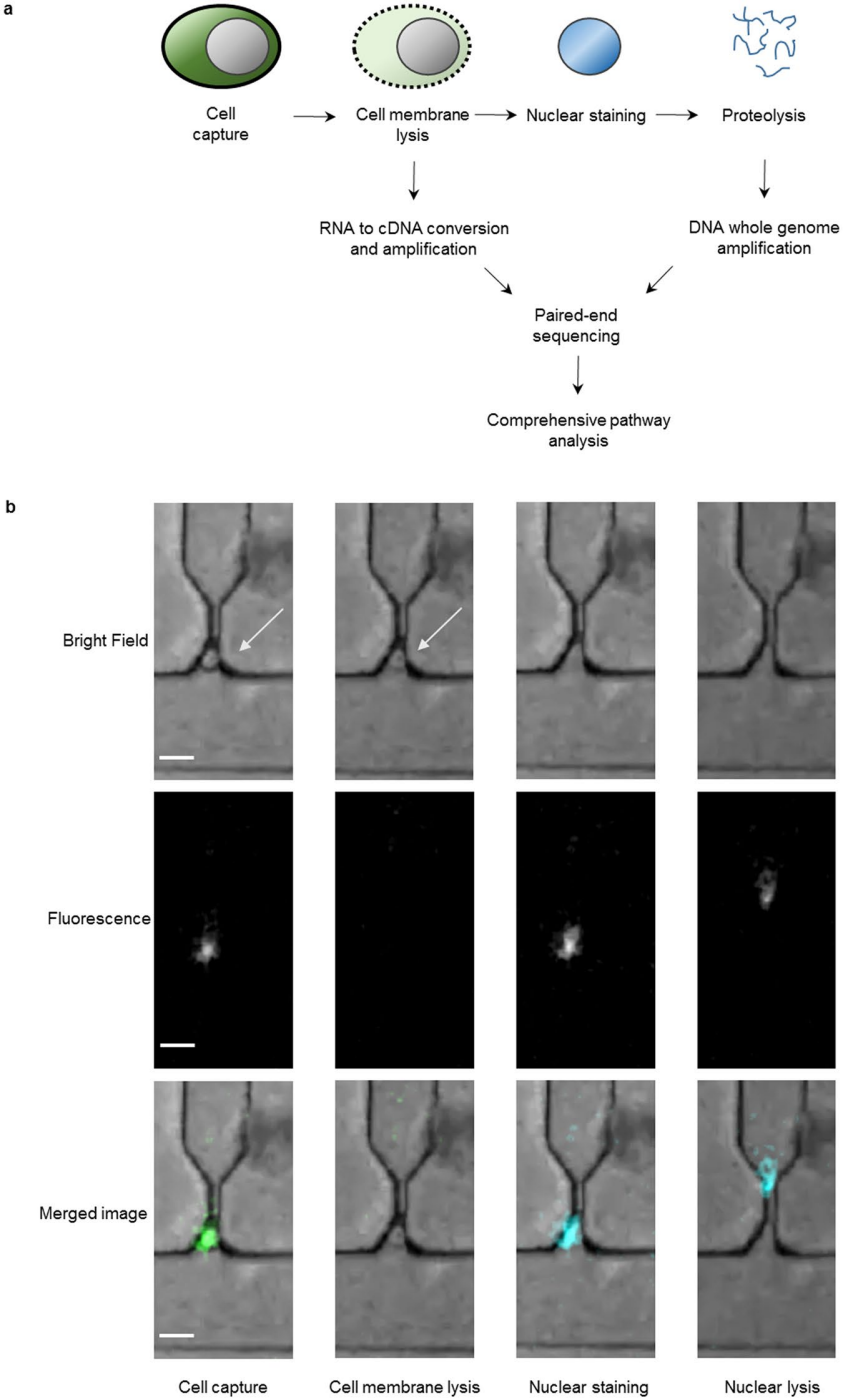
Two-stage lysis. (**a**) Diagram of the process flow: a single cell is trapped and its membrane is lysed releasing the mRNA that is collected. Next, the DNA is stained with fluorescent YOYO-1 dye to confirm its presence in the trap before it is lysed and genomic DNA can be collected in the outlet. (**b**) Bright field and fluorescence and merged time-lapse images from the instrument, during trapping, cell membrane lysis (loss of cytosol dye) and staining of the nuclear DNA retained in the cell trap. The scale bar corresponds to 15 *μ*m. The white arrow in bright-field image shows the position of the trapped cell. In the merged image the fluorescence is shown in colour.

Figure 2a shows various steps in the two-stage lysis protocol for separate RNA and DNA extraction using the microfluidic chip. First calcein-stained cells are captured in pl volume traps. Calcein fluorescence allows monitoring the presence and movement of these viable cells. Next the first lysis buffer (0.5x TBE containing 0.5% (v/v) Triton X-100 to which the DNA intercalating YOYO-1 dye is added) is applied. This buffer lyses the cell membrane, releasing the cytosol contents into the trap outlets filled with 10–20 *μ*l nuclease-free H_2_O, leaving the nucleus with the DNA in the trap. This process can be monitored as a time-lapse series (Fig. 2b), where the top row shows the bright-field, the middle row the fluorescent, and the bottom row the merged images. Through pseudo-colors, the calcein (green) and YOYO-1 (blue) fluorescence have been highlighted. The first column shows the situation with a trapped cell (see arrow). After application of the first lysis buffer, the fluorescence due to the calcein disappears (second column). After ≃3 minutes the fluorescence signal reappears as the YOYO-1 dye stains the DNA (third column). The cytosol content of each separate cell is lysed into and collected from a separate outlet and processed off-chip to synthesize and amplify cDNA. Subsequently, the nuclear lysis is performed with a second buffer (0.5x TBE containing 0.5% (v/v) Triton X-100 and Protease K). This causes the disappearance of the DNA from the traps into the outlets; see fourth column Fig. 2b. After reagent addition for whole genome amplification (WGA), the system is put at 30 °C for on-chip WGA. The final step is a 60 °C heat kill to end the amplification process. Subsequently, each sample is collected and sent with the corresponding cDNA sample for sequencing.

**Figure 3:**
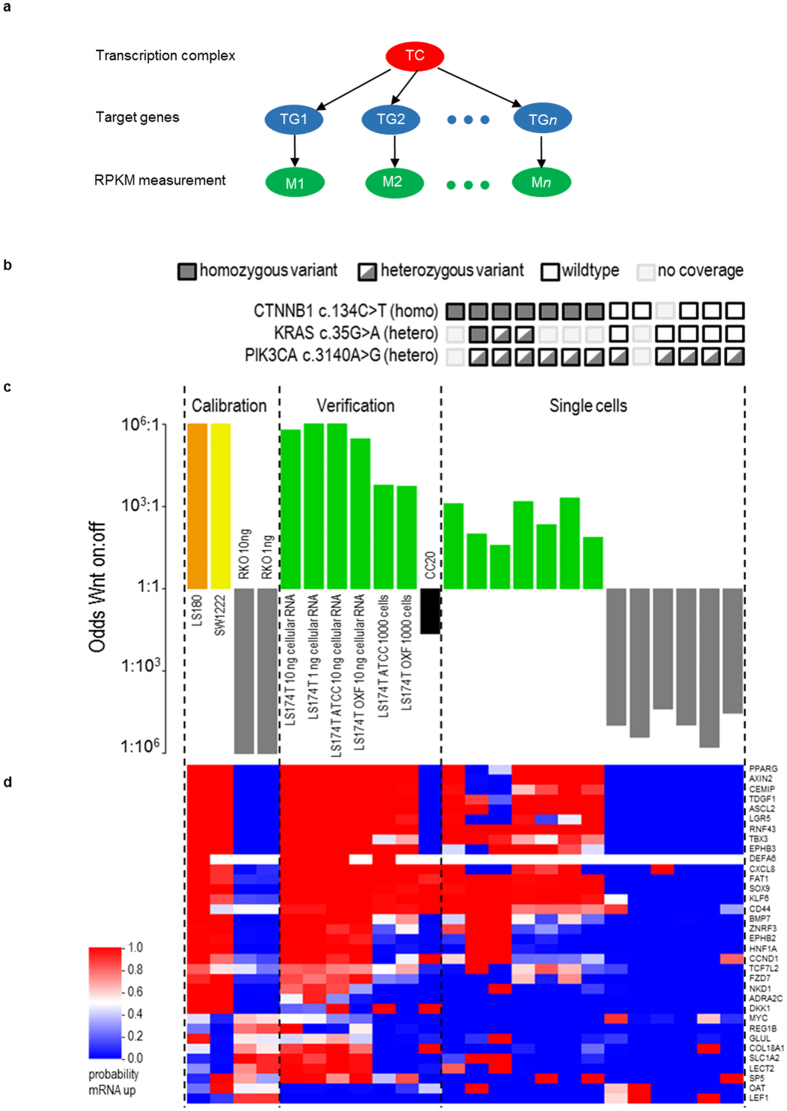
Complete Wnt pathway analysis results. (**a**) The structure of the Bayesian network used to model the transcriptional activity of a signal transductional pathway. (**b**) The panel shows putative driver mutations identified in the genes *CTNNB1*, *KRAS*, and *PIK3CA* for these samples, using the DNA derived from the same single cell for which the odds on Wnt activity are displayed in the corresponding columns below (**c**). (**c**) Results from Wnt pathway model analysis for the samples studied. Each sample result is represented by a bar with different colours for each cell line, as indicated. The y-axis shows the inferred odds of the Wnt pathway being active (on) versus inactive (off) on a logarithmic scale. For calibration, the RNA extracted from four colon cancer cell line experiments were used, which were known to be Wnt active (LS180, orange; SW1222 yellow) or inactive (RKO, grey) based on analysis of microarray data (see GSE 24795) and literature^17^. Subsequently the model was verified for LS174T (green) and a CC20 (black) samples known to be Wnt (in)active based on analysis of microarray data (see GSE 24795) and the literature^18^. Finally, the Wnt activity odds were inferred for the single cells processed: LS174T (green) and RKO (grey). All cells were correctly classified as Wnt active or inactive. (**d**) Cluster plot of the gene expression for the single cell data for LS174T and RKO cells including the expression data for bulk reference samples (LS180, SW122, RKO, LS174T and CC20) across the 34 genes constituting the Wnt profile, which are listed on the right hand-side^6^. The colour scale, as also shown on the left side, indicates the degree to which a gene’s expression is above (red) or below (blue) its threshold value set to separate the active from inactive calibration samples. See the main text for how the variation in the individual gene expressions translate into the resulting odds for the Wnt transcription complex activity i.e. Wnt pathway being (in)active.

Functional activity of the canonical Wnt pathway was analysed using a Bayesian network that represents its transcriptional program^6, 12^ with three types of nodes: (a) a transcription complex (TC), (b) Wnt target genes, and (c) expression level measurements of the target genes (Fig. 3a). The model *quantitatively* describes the relation between the TC and the target genes, and between each target gene and its associated measurements. Once such a model has been constructed from prior knowledge about which genes and expression levels to include, the model can be used on newly-analyzed samples by entering their measured expression values and applying Bayesian reasoning *to infer* the probability that the TC must have been active^6, 15^. The initial Bayesian Wnt pathway model, including 34 target genes, constructed using microarray data^6, 16^, is adapted to RNA-sequencing data here. The model was first calibrated on RNA-seq data from samples with known Wnt activity status ﻿based on analysis of microarray data (see GSE 24795) and literature^17^, using log_2_ RPKM measurements (reads per kilobase per million mapped reads): two Wnt active LS180 and SW1222 samples and two Wnt inactive RKO samples, shown as the first group in Fig. 3c.

Subsequently, the calibrated model was used on newly-analysed samples to infer the probability of TC activity. The model correctly classified CC20 and additional LS174T samples as Wnt inactive and Wnt active, respectively (labelled’verification’ in Fig. 3c), given previous microarray (see GSE24795) and literature data^18^. This verification also showed that the amount of RNA input (1 or 10 ng), did not influence the results (first two’verification’ samples in Fig. 3c). No differences in reported pathway activity were found for the LS174T cell lines, whether purified RNA extracted from bulk samples, or cells themselves (e.g. crude lysate) were directly used as input. This is relevant because in the single cell experiments, cells will serve as input rather than purified RNA. Finally, there was no difference in predicted pathway activity between cell line samples from the Weatherall Institute (Oxford) or those purchased from the ATCC; even when 3–4 cells were used as input (data not shown). Finally, RNA-seq measurements of single cells were analysed (‘single cell’ group in Fig. 3c). All seven analysed single LS174T cells (green) were classified to be Wnt active, showing that the Bayesian model can determine Wnt activity in single cells. These single cells were not synchronized in order to assess the power of our approach for determining pathway activity in a heterogeneous pool of cells. Figure 3d shows a cluster plot of the probability that the genes in the Bayesian Wnt model are up- or down-regulated compared to the threshold values set in the calibration step. Although RNA expression levels of individual genes varied between cells (see Fig. 3d), the model robustly infers Wnt pathway activity as it uses a panel of 34 target genes.

Some genes, up-regulated in LS174T verification samples with RNA as input (first four green bars), were not up-regulated when 1000 cells (with a similar RNA content) and single cells were used. These genes are generally expressed at low levels making them prone to disturbances in the amplification process, due to the presence of crude cell content. Still, from the calculated odds for the TC to be present in these samples (odds ratio ~1:1000), the Wnt pathway is active, despite some of these genes no longer being upregulated. The Bayesian Wnt model was constructed to give more weight to up-regulated genes in test samples, by a factor of approximately five, than non-up-regulated genes (see the probability Tables in Eq. (1)^6^) because of the intrinsic noise in biological systems. A number of confounding factors exist that may lower the expression of a gene even though the pathway and its transcription complex are active^19^. Bayesian modeling is very suited to deal with this noisiness. As a negative biological control we analysed single RKO cells (grey), known to be Wnt inactive based on microarray data (see GSE24795). As Fig. 3c shows, all 6 cells studied were inferred to be Wnt inactive. Our pathway model thus correctly classified all 7 Wnt active and 6 Wnt inactive validation samples (Fisher’s exact test p = 0.0006).

**Figure 4:**
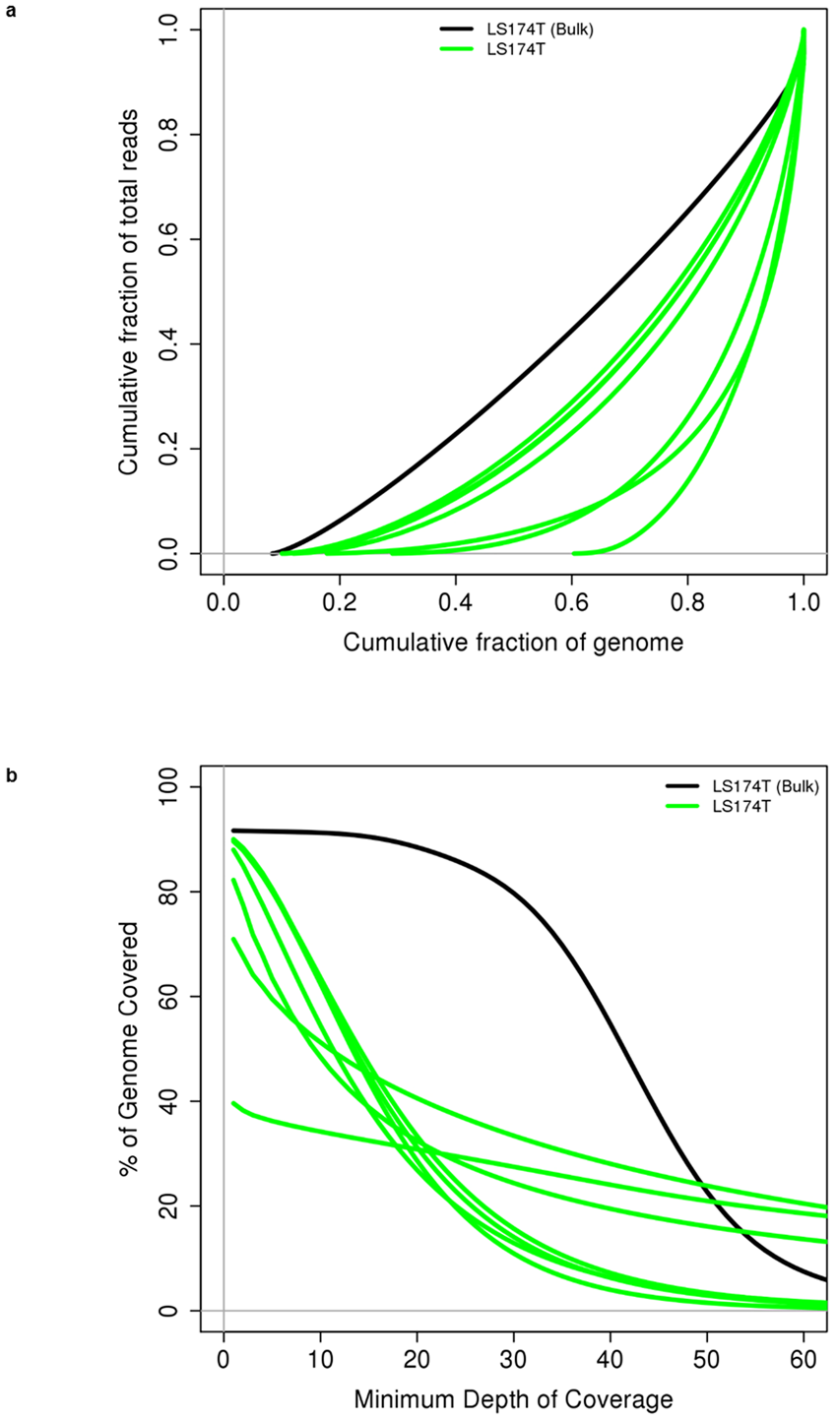
Lorenz and coverage graphs. (**a**) Lorenz graph and (**b**) coverage graph of the DNA sequencing coverage for the single cells samples LS174T (green lines) and a bulk LS174T sample (black) shown as reference.

For the same cells, DNA isolated and amplified on chip was sequenced. Figure 4 shows the Lorenz (a) and coverage graphs (b) obtained for this data. The Lorenz graph in Fig. 4a displays the cumulative fraction of total reads as a function of the cumulative fraction of the genome covered. The advantage of a Lorenz graph is that it provides a good representation of the coverage diversity even at low coverage. In our initial experiments the percentage of non-covered bases was high and varying. After improving the temperature regulation, the percentage of non-covered bases could be reduced to 10.6 ± 0.9% for an average read depth of 16.2 ± 1.0, which is close to the 8.4% non-covered bases obtained for a bulk sample (see Supplementary Table S1), which is important for driver mutation identification. Moreover, this also compares favourably to previous single cell sequencing methods. For the multiple annealing and looping-based amplification cycles (MALBAC) method, coverages of 85 up to 93% (≥1x) were achieved^20^. Other (bench-based) single cell RNA and DNA sequencing methods yield a coverage between 62–80% (at 0.6–2.5x) for DR-seq^13^ and 67.2 ± 8.1% for G&T-seq using multiple-displacement amplification (MDA)^14^. Note that in some publications these numbers are provided in comparison to the bulk coverage^20^. Therefore our results show the advantage of the on-chip DNA amplification for single cell DNA analysis to improve coverage. Figure 4b shows the coverage graph for these data i.e. the % of the genome covered vs the coverage depth. This graph is useful as it shows the percentage of the bases that achieve the 20 or 30x coverage typically used for SNP calling. Note that further sequencing will shift the fraction of the bases with sufficient coverage, but it will not improve the fraction of the bases covered, as this is determined by the initial loss of DNA going into the first amplification step.

From a mutation analysis of the DNA sequencing data for samples with good coverage, C ≥ 70% of the genome, the known driver mutation in *CTNNB1* (c.134 C > T) for the Wnt pathway in the LS174T cell line was found, see Fig. 3b. This *CTNNB1* mutation leads to an amino acid change S45F, which alters potential GSK-3 *β* phosphorylation sites of *β*-catenin, thereby hampering its degradation and hence leading to activation of the TCF4/*β*-catenin transcription complex driving the canonical Wnt pathway^21^. Also other known mutations of LS174T cells in *KRAS* (c.35 G > A) and *PIK3CA* (c.3140 A > G) were found, see Fig. 3b. A mutation analysis of RKO single cells showed wild type *CTNNB1* in 5 out of 5 cells (one had insufficient coverage), as expected for RKO and matching the Wnt pathway inactivity. The RKO single cell DNA analysis showed wild type *KRAS* in 5 out of 5 cells, and mutated *PIK3CA* in 5 out of 5 cells. The latter mutation (c.3140 A > G) induces activity of the PI3K pathway in the RKO cells, and also in the LS174T cells, as indicated by an inactive FOXO transcription factor, even though we found FOXO family members are expressed.

## Discussion

Two other approaches have been published for analysing both the genome and transcriptome from the same single cell, namely DR-seq^13^ and G&T-seq^14^ recently. These are bench protocols which can be automated by using a robotic workstation^14^. By contrast, the approach we have presented here is an *integrated* approach, in which the two crucial process steps, RNA and DNA separation and single cell DNA amplification, have been performed in a microfluidic chip. A physical method of RNA and DNA separation is implemented using a microfluidic chip and a two-stage lysis protocol. Compared to the DR-seq approach, the use of a physical method to separate the gDNA from the RNA has three advantages. First, there is no need for masking-out the coding sequences when mapping the gDNA fraction to the genome^13^, implying that mutations in genes which are not expressed or are suppressed can also be found. Secondly, the whole cytosol and nuclear extract is used, while in the DR-seq approach the sample is split in half^13^. Thirdly, conventional, commercial kits for single cell RNA and DNA amplification can be used, as the two nucleic acids fractions are separated, whereas DR-seq requires an additional *in-vitro* transcription step in the RNA sample preparation^13^. The G&T-seq method does use physical means for separating the RNA and DNA by using oligo-dT coated magnetic beads to extract the mRNA. Here the advantage of our approach is that the processing is done within a microfluidic chip, which limits handling, transfer and separation steps and thereby reduces sample loss and contamination. This is particularly important, when processing the DNA and RNA content from a single cell^13^. Note that the G&T-seq protocol involves a number of handling steps to separate the RNA and DNA. To maximize gDNA recovery, the beads capturing the RNA have to be washed four times and the eluate of each wash has to be added to the original supernatant with the gDNA. Finally, a microfluidic cartridge in which processing is done may also bring cost benefits through reduction of labor and use of standard commercial kits, which should outweigh the costs of the dedicated instrument required.

The RNA-seq data have been analyzed based on a Bayesian model for the canonical Wnt pathway, known to be active in colorectal cancer (cell lines)^16^. Similar Bayesian models are being developed for the other tumor driving pathways. Once these additional models become available, one could determine which tumor driving pathways are active from the measured gene expression profiles, without prior knowledge which pathway may be active. Since tumors tend to be heterogeneous and comprised of (sub)clones, this work would enable a systematic approach to find these clones, by first establishing which tumor driving pathways are active and then finding the mutations that drive them, using single cell resolution. This approach can be seen as an example of an integrative network modelling approach (see refs 22 and 23). Being able to see which tumor driving signalling pathways are active as the disease progresses, may provide new insights into tumor evolution, and may enable the selection of targeted drug combinations to treat cancer patients more effectively.

## Conclusion

We have presented a method using a microfluidic chip and a two-stage lysis protocol, to extract both the RNA and the DNA from the same single cell. An important advantage of this approach is that the DNA and RNA content from a single cell can be processed directly after lysis, owing to the picoliter cell trap volume, without the need for additional washing and handling steps that increase the risk of removing some of the nucleic acid content. Moreover, in contrast to the G&T method, our method will also extract RNAs without a poly-A tail^24^, and thus could enable the partitioning of any RNAs between cytosol and nucleus^25, 26^.

Sequencing of each fraction enables determination of pathway activity from the cytosol faction and the mutations responsible for driving this pathway from the nuclear fraction. This ability to predict pathway activity indicates that the Bayesian pathway-modelling approach, previously only applied to bulk samples, can be extended to single cells. Identifying active pathways and their driver mutations and weighing their contribution by single cell analysis of a heterogeneous tumour could be important for guiding targeted therapy. Pathway analysis by differential on-chip DNA and RNA extraction could also be a very promising approach for analysis of individually captured circulating tumour cells.

## Methods

### System

The set-up used to monitor and control single cells in the microfluidic chip and enabling movement, trapping, RNA/DNA extraction and amplification comprises the following modules:

- A microscopy module enabling epi-fluorescence and bright-field imaging
- A temperature module
- A flow control module
- Microfluidic chip adaptor and tube connector
- XY translation and focus adjustment unit

Each module is operated through a common graphical user interface program (GUI) and characterized as follows (see Fig. 1):

*a) Microscopy module*: The main elements of the module are the following components: Microscope objective (Wild Heerbrugg: 10X, NA 0.45). Excitation light source for fluorescence microscopy: A LED with center wavelength at 470 nm (Thorlabs model M470L3) equipped with collimator lenses. Fluorescence filters (Semrock, barrier filter model 733–527/645–25, dichroic mirror model 733–474/23–25, excitation filter model 733–495/605-Di01-25 × 36). CMOS imaging sensor (Fairchild, CIS1910) with 1920 × 1080 pixels, and pixel size 6.5 *μ* m, using a home-built image board. The bright-Field illuminator delivers light from a LED with center wavelength of 505 nm.

*b) XY translation and focus adjustment unit*: The XY translation is managed by moving the microfluidic chip in the Y-direction and illuminator-objective arm in the X-direction. The z-translation for focussing is performed by moving the objective along the optical axis.

*c) Flow control module*: The control of moving and trapping the cells in the microfluidic chip is achieved by applying minor elevated air pressure of typically 5–10 mbar onto the inlet channels. The pressure is delivered by an external pressure-based flow control module (Fluigent, MFCSTM–EZ 4C). This provides 4 pneumatic, independently controllable channels that are connected to the tube connector of the chip adaptor, see Fig. 1a and b.

*d) Temperature module*: The heating and cooling of the microfluidic chip is done by three Peltier elements. The temperature profile used for the nucleic acid amplification within the chip is regulated by PID control of the Peltier elements. Temperature feedback is provided by a thermo-couple situated inside the heating /cooling plate that the microfluidic chip is clamped on (see Fig. 1). Due to a temperature difference between this plate and the sample volume, the plate temperature is elevated by a calibrated offset.

#### Device fabrication

30 *μ*m deep microchannels were created by reactive ion etching in silicon and replicated in nickel by electroplating. The nickel master was replicated in TOPAS 5013 (Topas, Germany) by injection molding using a tool creating twelve holes for fluidic connections. The device was sealed by UV-assisted thermal bonding of a 150 *μ*m thick TOPAS 5013 foil (Topas, Germany).

#### Cell culture

The colorectal cancer cell line samples came from the Weatherall Institute of Molecular Medicine (Oxford, U.K.), which maintains a large collection of colorectal cancer cell lines. The cell lines CC20, LS174T, LS180, and SW1222, were cultured in DMEM (Gibco, Life Technologies). RKO was cultured in RPMI (Gibco, Life Technologies) all media were supplemented with 10% FBS (Gibco, Life Technologies), 2 mM Glutamax (Life Technologies) and 1% pen/strep (Gibco, Life Technologies). Cells were grown at 37 °C in a humidified incubator containing 10% CO_2_. Prior and after experiments, the cells were tested for Mycoplasma contamination (Agilent). For verification purposes an additional LS174T cell line sample was purchased directly from the ATCC (Manassas, VA, USA).

#### Cell staining

Freshly prepared cells were detached with 0.25% trypsin containing 0.04% EDTA (Gibco). Upon detachment the cells were taken up in medium and spun down, the cells were washed once with PBS and finally resuspended in PBS containing 1 *μ*M calcein-AM (Sigma Aldrich). Cells were incubated for 30 minutes at room temperature prior to loading them onto the chip. The on-chip nuclear staining was achieved during lysis of the cellular membrane by applying a lysis buffer consisting of 0.5x TBE (Sigma Aldrich) containing 0.5 % Triton-X100 (Sigma Aldrich) and 1 *μ*M YOYO-1 (ThermoFischer Scientific). (See main text and Fig. 2).

#### RNA extraction

RNA was extracted and purified from the cell line samples described in the cell culture section using a commercial kit (VERSANT Sample Preparation 1.0 Reagents kit) according to manufacturer’s instructions. The cells were trypsinized, washed once with PBS and resuspended in PBS. RNA was extracted from ≈10^5^ cells and stored at −80 °C.

#### Sample preparation

The RNA fraction of a single cell was converted to cDNA and amplified using the SMART-Seq v4 Ultra Low Input RNA Kit for Sequencing (Takara/ Clontech) according to manufacturer’s instructions^27^. For the calibration and verification of the pathway model also higher inputs of cells or purified RNA were used for cDNA synthesis and amplification using the same kit. The gDNA fraction of a single cell was amplified on chip using the REPLI-g Single Cell kit (Qiagen).

#### Sequencing

Sequencing libraries were prepared using the Nextera libray preparation kit from inputs as low as 10 ng. The Nextera XT DNA library preparation kit was used when the input was only ≥1 ng of cDNA or DNA. After cluster formation on the Illumina cBot using the HiSeq Paired End Cluster Kit v4, sequencing was done on the Illumina HiSeq. 2500, using 2 × 125 bp paired-end sequencing with the HiSeq SBS kit v4 (250 cycles).

#### Bioinformatic analysis

The DNA sequencing reads were aligned to the reference human genome GRCh37 release 60 using the Burrows-Wheeler alignment tool version 0.7.5a^28^. Reference coverage statics were computed with the BEDtool version 2.9.0 on the bam files.

For alignment of the RNA sequencing data the pipeline proposed by Fonseca *et al*.^29^ was used (version 0.4.2, mapper = tophat2 (v2.0.10), quantification method = htseq. 2 (v0.6.1p1)). Determination of the gene expression levels was described in reads per kbase per million mapped reads, RPKM.

## Electronic supplementary material

Supplementary Information
