# Supplementary material for: Complete sequence-based pathway analysis by differential on-chip DNA and RNA extraction from a single cell
Source: Sci Rep. 2017 Sep 8;7:11030. doi: 10.1038/s41598-017-10704-4 (PMC5591254; doi:10.1038/s41598-017-10704-4)
Supplement: Supplementary file 1 — Supplementary Information [file 41598_2017_10704_MOESM1_ESM.pdf]

Supplementary information to Complete sequence-based pathway analysis by differential on-chip DNA and RNA extraction from a single cell

D. van Strijp[1], R.C.M. Vulders[1], N.A. Larsen[2], J. Schira[3], L. Baerlocher[3], M.A. van Driel[1], M. Pødenphant[4], T. Hansen[2], A. Kristensen[4], K.U. Mir[5], T. Olesen[2], W.F.J. Verhaegh[1], R. Marie[4], and P.J. van der Zaag[1,*]

[1] Philips Research Laboratories, High Tech Campus 11
5656 AE Eindhoven, The Netherlands
[2] Philips BioCell, Gydevang 42,
3450 Allerød, Denmark
[3] Fasteris SA, Chemin du Pont-du-Centenaire 109
CH-1228 Plan-les-Ouates, Switzerland
[4] DTU Nanotech, Ørsteds Plads Building 345 east
2800 Kgs. Lynby, Denmark
[5] XGenomes, Pagliuca Harvard Life Lab, 127 Western Avenue
Boston, MA 02134, U.S.A.
* e-mail: p.j.van.der.zaag@philips.com
phone: +31-40-22749481 fax: +31-40-2742944

Keywords
single cell analysis, pathway analysis, RNA and DNA extraction, sequencing

Supplementary information:

Caption Supplementary Table

Table S1 *Sequencing coverage for the single cell DNA sequencing data*
The sequencing coverage for the single cell DNA sequencing results compared to a bulk reference sample.

Supplementary Table 1. Sequencing coverage for the DNA single cell sequencing data.

Cell line	# Mapped bases (10^9)	Mean coverage	Median coverage	% not covered
LS174T (bulk)	127.9	41.3	40	8.36
LS174T	102.6	33.1	8	17.8
LS174T	117.9	38.1	10	29.1
LS174T	102.3	33.1	0	60.4
LS174T	46.5	15.0	12	10.0
LS174T	51.9	16.8	13	10.4
LS174T	45.6	14.7	10	12.0
LS174T	50.0	16.2	13	10.1
RKO	37.7	12.2	3	28.5
RKO	38.5	12.4	2	37.1
RKO	36.6	11.8	8	12.0
RKO	36.9	11.9	8	11.4
RKO	35.5	11.5	9	10.7
RKO	42.6	13.8	8	13.3
